# Triple Therapy with Metformin, Ketogenic Diet, and Metronomic Cyclophosphamide Reduced Tumor Growth in MYCN-Amplified Neuroblastoma Xenografts

**DOI:** 10.3390/metabo13080910

**Published:** 2023-08-03

**Authors:** Luca Catalano, Sepideh Aminzadeh-Gohari, Daniela D. Weber, Rodolphe Poupardin, Victoria E. Stefan, William J. Smiles, Julia Tevini, René G. Feichtinger, Sophia Derdak, Martin Bilban, Stefan Bareswill, Markus M. Heimesaat, Barbara Kofler

**Affiliations:** 1 Research Program for Receptor Biochemistry and Tumor Metabolism, Department of Pediatrics, University Hospital of the Paracelsus Medical University, 5020 Salzburg, Austria; l.catalano@stud.pmu.ac.at (L.C.);; 2 Spinal Cord Injury and Tissue Regeneration Center Salzburg (SCI-TReCS), Cell Therapy Institute, Paracelsus Medical University, 5020 Salzburg, Austria; 3 Core Facilities, Medical University of Vienna, 1090 Vienna, Austria; 4 Department of Laboratory Medicine, Medical University of Vienna, 1090 Vienna, Austria; 5 Gastrointestinal Microbiology Research Group, Institute of Microbiology, Infectious Diseases and Immunology, Charité-University Medicine Berlin, Corporate Member of Free University Berlin, Humboldt University Berlin and Berlin Institute of Health, 12203 Berlin, Germany

**Keywords:** neuroblastoma, ketogenic diet, metformin, MYCN, β-oxidation

## Abstract

Neuroblastoma (NB) is a childhood cancer in which amplification of the MYCN gene is the most acknowledged marker of poor prognosis. MYCN-amplified NB cells rely on both glycolysis and mitochondrial oxidative phosphorylation (OXPHOS) for energy production. Previously, we demonstrated that a ketogenic diet (KD) combined with metronomic cyclophosphamide (CP) delayed tumor growth in MYCN-amplified NB xenografts. The anti-diabetic drug metformin (MET) also targets complex I of the OXPHOS system. Therefore, MET-induced disruptions of mitochondrial respiration may enhance the anti-tumor effect of CP when combined with a KD. In this study, we found that MET decreased cell proliferation and mitochondrial respiration in MYCN-amplified NB cell lines, while the combination of KD, MET, and low-dose CP (triple therapy) also reduced tumor growth and improved survival in vivo in MYCN-amplified NB xenografts. Gene ontology enrichment analysis revealed that this triple therapy had the greatest effect on the transcription of genes involved in fatty acid ß-oxidation, which was supported by the increased protein expression of CPT1A, a key mitochondrial fatty acid transporter. We suspect that alterations to ß-oxidation alongside the inhibition of complex I may hamper mitochondrial energy production, thus explaining these augmented anti-tumor effects, suggesting that the combination of MET and KD is an effective adjuvant therapy to CP in MYCN-amplified NB xenografts.

## 1. Introduction

Neuroblastoma (NB) is an embryonal tumor derived from progenitor cells of the sympathetic nervous system [1]. Although NB accounts for only 6% of all pediatric cancer cases, whereby the 5-year survival rate is 81% [2], there is still a subpopulation of high-risk patients with a 5-year event free survival (EFS) of approximately 50% [3]. Almost half of these patients harbor an amplification of the *MYCN* oncogene, which is the most well-characterized marker of poor prognosis in NB [4]. However, therapeutic strategies aimed at direct or indirect targeting of MYCN are not clinically available at present [5].

Tumor cells become metabolically reprogrammed to sustain the biomass and energy production required for rapid cellular growth [6]. MYCN has been shown to increase these growth demands by upregulating fundamental metabolic pathways such as glycolysis, oxidative metabolism, and lipogenesis, and the metabolism of glutamine, serine, and glycine [7,8,9]. Such alterations highlight the ability of cancerous cells to hijack cellular metabolism to cater to growth and proliferation. Indeed, cancer cells not only rely heavily on glucose metabolism, a phenomenon known as the Warburg effect, but frequently possess appreciable metabolic ‘flexibility’ [10]. The latter confers to cancer cells the capability to leverage alternate metabolic pathways to gain a survival advantage in the face of stress arising from the hypoxic and nutrient-deprived tumor microenvironment [11]. This may explain why some patients fail to respond to, or relapse following, intense multi-modal therapy [12]. In that regard, targeting metabolic vulnerabilities induced by MYCN may represent an innovative and otherwise unexplored approach to tackle high-risk NB.

A very low carbohydrate, low protein, and high fat ketogenic diet (KD) has emerged as a potential therapeutic intervention in cancer, with several pre-clinical studies demonstrating an anti-tumor effect, especially when combined with conventional cytotoxic therapies [13]. Indeed, the KD appears to have the most beneficial effect in cancer treatment as an adjuvant therapy [14]. KDs have the potential to ‘rewire’ multiple metabolic pathways simultaneously. Low glucose availability combined with elevated ketone bodies are candidate metabolic variables to drive the anti-proliferative effect of KDs, inter alia, brain tumors [15,16]. In addition, KDs have been shown to alter amino acid and lipid metabolism [13,17]. Yang and colleagues demonstrated that a KD enhanced the anti-tumor effect of classical chemotherapy in pancreatic cancer, conferring a survival advantage associated with a higher NADH/NAD ratio in tumor cells. This suggests alterations to the redox state as a potential contributor to the anti-tumor effect of the KD when combined with chemotherapy [18]. We previously reported that a KD in combination with CP reduced tumor growth in NB xenografts, but failed to induce complete remission [19].

Targeting the oxidative phosphorylation (OXPHOS) system has been shown to be a potential therapeutic approach in cancer treatment [20,21]. Given the emerging role of OXPHOS in MYCN-amplified NB metabolism [8], exploiting its inhibition may represent a rational therapeutic approach for NB treatment. Metformin (MET) is a common anti-diabetic drug that targets mitochondrial respiration by inhibiting complex I (NADH dehydrogenase) of the OXPHOS system [22,23,24]. The inhibition of complex I has been demonstrated to reduce tumor growth in divergent tumor xenograft models [22,25]. In this regard, MET alone was also shown to suppress tumor growth in MYCN-amplified and non-amplified NB-bearing mice [26]. Therefore, the aim of this study was to determine whether MET could enhance the efficacy of a KD combined with low-dose CP in MYCN-amplified NB xenografts. To elucidate the mechanism by which the combination of KD, CP, and MET (triple therapy) reduced tumor growth, we measured plasma pro-inflammatory factors and metabolic biomarkers potentially involved in cancer progression. Then, we performed RNA-seq analysis to reveal alterations at the transcriptional level, with a view to uncover the anti-tumor mechanism effect of the therapy.

## 2. Materials and Methods

### 2.1. Cell Lines

*MYCN*-amplified NB cell lines SKNBE(2) (CRL-2271, ATCC, Manassas, VA, USA) and KELLY (92110411, Sigma-Aldrich, Darmstadt, Germany) were selected for cell proliferation and xenograft studies. The cells were cultured as previously described in a standard medium containing a 1:1 mixture of Eagle’s Minimum Essential Medium (M5650, Sigma-Aldrich, Irvine, UK) and Ham’s F12 (N4888, Sigma-Aldrich, Irvine, UK), supplemented with 10% fetal bovine serum (10500064, Gibco, Paisley, UK), Glutamax (35050038, Thermo Fisher Scientific, Paisley, UK), MEM Non-essential amino acid solution (M7145, Sigma-Aldrich, Irvine, UK), and penicillin/streptomycin/amphotericin (US17-745E, Lonza, Basel, Switzerland) [27]. The cell lines were tested for mycoplasma contamination, and were maintained at 37 °C with 5% CO_2_.

### 2.2. Cell Proliferation Assays

The MET (PHR1084, Sigma-Aldrich, Laramie, WY, USA) stock solution was prepared in standard NB cell culture medium. The acetyl CoA carboxylase (ACC) inhibitor PF 05175157 (5790, Tocris, Bristol, UK) was dissolved in DMSO and stored at −80 °C. 1.9 × 10^4^ cells/well of SKNBE(2) cells and 8 × 10^3^ cells/well of KELLY cells were seeded in 96-well plates. Then, the cells were treated with different concentrations of MET (1 to 20 mM) for 72 h, or with PF 05175157 (3–33 µM) for 48 h. Standard medium was used as a vehicle control of the MET-treated cells, whereas DMSO was used as a vehicle control of the PF 05175157-treated cells. Cell viability was measured via a crystal violet assay, as previously described [28].

### 2.3. Bioenergetic Analysis

For the bioenergetic analysis, 1.4 × 10^3^ cells/well of SKNBE(2) and 7 × 10^3^ cells/well of KELLY cells were seeded in Seahorse XF cell culture microplates (102601-100, Agilent, Santa Clara, CA, USA) using standard cell culture medium. The NB cells were cultured for 72 h and subsequently treated with varying concentrations of MET (1–10 mM) for 24 h. Alternatively, the NB cells were grown for 24 h following seeding, and then treated with PF 05175157 (3 µM to 33 µM) for 72 h. On the day of the experiment, the medium was changed to Seahorse assay medium consisting of XF DMEM (103575-100, Agilent, Santa Clara, CA, USA) supplemented with 1 mM pyruvate, 10 mM glucose (Agilent, Santa Clara, CA, USA) and 2 mM glutamine. The Seahorse XF Cell Mito Stress Test was then performed according to the manufacturer’s protocol and specifications of the Seahorse XFe96 Analyzer (Agilent, Santa Clara, CA, USA). Following the assay, the Seahorse XF cell culture microplates were placed at −80 °C, and the data was normalized to the nucleic acid content using a CyQUANT cell proliferation assay (C7026, Thermo Fisher, Eugene, OR, USA) in accordance with the manufacturer’s protocol.

### 2.4. Animal Models and Sample Preparation

The animals were maintained under specific pathogen-free conditions in accordance with the Salzburg Animal Care and Use Committee (20901-TVG/115/21-2020) and the Austrian Federal Ministry of Education, Science and Research (BMBWF) (2021-0.711.738). Xenografts were established by injecting 200 µL of a 1:1 mixture of NB cells (2 × 10^7^ cells per mouse) in serum-free medium and Matrigel (356234, Corning, New York, NY, USA) in the right flank of 5- to 6-week-old female CD-1 nude mice (Charles River, Sulzfeld, Germany). Once the tumor reached ~100 mm^3^, the tumor-bearing mice were randomized into the different therapy groups (*n* = 6–10). The tumor-bearing mice were group-housed, and received *ad libitum* access to food and water. The tumor volumes were measured twice weekly using a caliper, which was calculated with the formula width × height × length/2. The body weight was monitored two times per week, and the blood glucose and ketone bodies (β-hydroxybutyrate) were assessed once per week using the enzyme-based Precision Xceed System (Abbott, Chicago, IL, USA). The mice were anesthetized once the tumor volume reached ~1500 mm^3^, or when the termination criteria based on the health status were reached. To calculate the overall survival, the SKNBE(2) xenografts were kept for a maximum of 33 days, and the KELLY xenografts were kept for 36 days. Then, the mice were euthanized, and plasma and tissue samples were collected as previously described [27].

### 2.5. Diet Composition and In Vivo Treatments

The mice were randomly assigned to the different therapy regimens. All of the diets were provided *ad libitum*. The diets were ordered from Ssniff and fortified with specific amounts of vitamins and mineral supplements to administer the same quantity to each therapy group. Detailed information on the diet composition and energy content is presented in Supplementary Table S1. As we previously reported, the food intake of CD-1 nude mice fed with a KD *ad libitum* was approximately half compared to mice fed with a control diet [27]. For this reason, although the diets were not isocaloric, the mice consumed comparable amounts of energy. CP (C0768, Sigma-Aldrich, Darmstadt, Germany) was administered via drinking water, according to the study by Man and colleagues [29]. The CP dose was selected to induce a mild but not significant effect on tumor growth, in order to enable the evaluation of any additive or synergistic effects of the different therapeutic interventions. The SKNBE(2) xenografts were treated with 13 mg/kg/day according to our previously published protocol [30], and KELLY xenografts were treated with 20 mg/kg/day (Figure S1). The selected dose of MET (100 mg/kg) was based on dose response experiments initially performed in the SKNBE(2) and KELLY xenografts (Figure S2).

### 2.6. Western Blot (WB) Analysis

SKNBE(2) xenograft tissue (20–30 mg) was homogenized with an Ultra-Turrax homogenizer (IKA) in 300 µL of RIPA lysis buffer (MFCD02100484, Sigma-Aldrich, St. Louis, MO, USA) supplemented with protease/phosphatase inhibitors (A32961, Thermo Scientific, Rockford, IL, USA) and 1 mM EDTA. The homogenates were centrifuged at 4 °C for 10 min, and the supernatant was collected. A total of 15 µg of protein was used for Western blot analysis. The primary antibodies were diluted in tris-buffered saline 0.5% Tween-20 (TBS-T) and 10% blocking reagent (11921673001, Roche, Mannheim, Germany). The following antibodies were used: heat shock protein A6 (HSPA6) (1:1000, 13616-1-AP, Proteintech, Wuhan, China), carnitine palmitoyltransferase 1A (CPT1A) (1:1000, 12252, Cell Signaling, Danvers, MA, USA), voltage-dependent anion channel (VDAC) (1:1000, ab15895, Abcam, Cambridge, UK), and β-actin (1:2000, ab8227, Abcam, Cambridge, UK). Horseradish peroxidase-labelled secondary antibodies were used (K400311-2, Dako, Glostrup, Denmark). The band densitometry was calculated using Image Lab Software 5.2.1 (Bio-Rad, Hercules, CA, USA) and normalized to β-actin.

### 2.7. Determination of Pro-Inflammatory and Metabolic Peptides in Plasma

The plasma levels of the murine pro-inflammatory factors (Table 1A) and metabolic peptides (Table 1B) were measured using V-plex and customized U-plex assays from Meso Scale Discovery (MSD, Rockville, MD, USA), according to the manufacturer’s instructions (Table 1). In the V-plex plate, the samples were measured in duplicate, whereas in the U-plex assay, only one replicate per sample was measured.

### 2.8. Molecular Analysis of the Microbiota from Fecal Content

DNA was extracted from the colon fecal content of NB-bearing mice, as described previously [31]. The total DNA was measured using Quant-iT PicoGreen reagent (Thermo Fisher, Eugene, OR, USA) and adjusted to 1 ng/µL. Specific 16S rRNA primers (Tib MolBiol, Berlin, Germany) were used to identify different species, genera, or groups of bacteria via qPCR [32,33]. The overall gene copy numbers per ng of DNA were calculated.

### 2.9. RNA Extraction, Sequencing, and Data Analysis

RNA was extracted from the SKNBE(2) xenograft tissue using the AllPrep DNA/RNA/Protein Mini Kit (80004, Qiagen, Germantown, MD, USA) according to the manufacturer´s instructions. Six samples per therapy group were randomly selected. Sequencing libraries were prepared at the Genomics Core Facility of the Medical University of Vienna using the NEBNext Poly(A) mRNA Magnetic Isolation Module, and the NEBNext UltraTM II Directional RNA Library Prep Kit for Illumina according to manufacturer’s protocol (New England Biolabs, Ipswich, MA, USA). The libraries were QC-checked on a Bioanalyzer 2100 (Agilent, Santa Clara, CA, USA) using a high sensitivity DNA kit for correct insert size, and quantified using a Qubit dsDNA HS Assay (Invitrogen, Eugene, OR, USA). The pooled libraries were sequenced on two flow cells of a NextSeq500 instrument (Illumina, San Diego, CA, USA) in 1 × 75 bp single-end sequencing mode. Per sample, an average of 27 million reads were generated. Reads in a fastq format were generated using the Illumina bcl2fastq command line tool (v2.19.1.403), including trimming of the sequencing adapters. Since the samples were human xenografts inoculated in mice, an additional stringent pre-alignment was performed where all of the reads in fastq format were aligned to the murine reference genome version GRCm38/mm10 using the STAR aligner [34] version 2.6.1a in 2-pass mode, in order to computationally remove reads that originated from the mouse portion in the tissue sample. Reads that remained unmapped in the pre-alignment step were recovered and aligned to the human reference genome version GRCh38 with Gencode 29 annotations using the STAR aligner, version 2.6.1a, in 2-pass mode. The raw reads per gene were counted with STAR [34]. The differential gene expression was calculated using DESeq2 version 1.22.2 [35]. The transcripts per million (TPM) were generated by RNA-Seq with Expectation Maximization (RSEM) [36]. The genes were considered significantly differentially transcribed with an adjusted *p*-value of <0.05. Then, we conducted gene ontology (GO) enrichment analysis using the ClusterProfiler R package v4.8 [37]. Pathways were considered significantly enriched when *p* < 0.05.

### 2.10. Statistics

Statistical analysis was performed using GraphPad Prism 9.0.0. The group variations are indicated as mean ± SD. The group differences were considered significant at *p* < 0.05. We performed a differential gene expression analysis with the DESeq2 package in R to identify genes that were significantly deferentially expressed between our experimental conditions. The DESeq2 package uses a model based on the negative binomial distribution to estimate the variance–mean dependence in count data, and tests for differential expression using the Wald test. Genes with an adjusted *p*-value < 0.05 (Benjamini & Hochberg) were considered significantly deferentially transcribed.

We conducted our enrichment analysis with the R package ClusterProfiler using Fisher’s exact test. GO or pathways were considered significantly enriched with a *q*-value < 0.05.

## 3. Results

### 3.1. Effect of MET on Respiration and Proliferation of MYCN-Amplified NB Cell Lines

**Figure 1:**
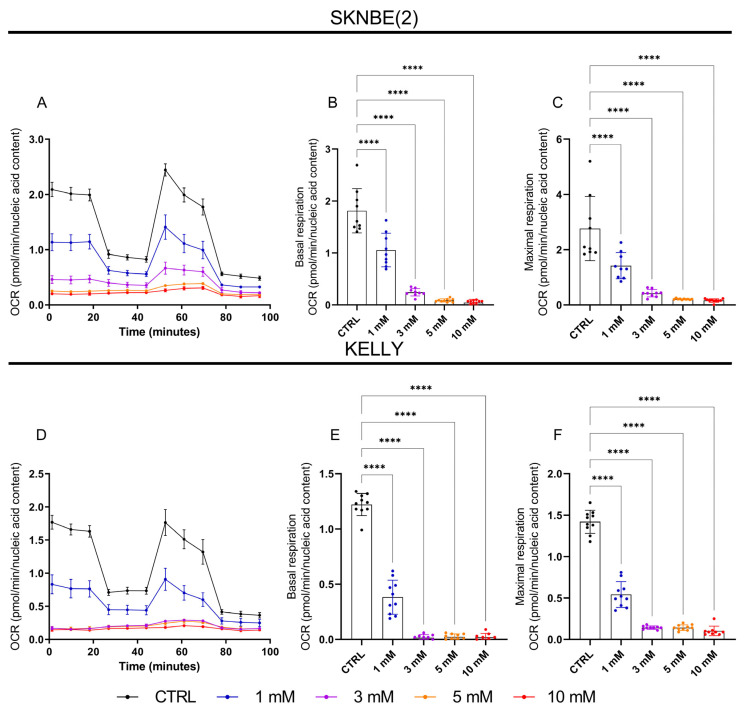
Effect of MET on oxygen consumption rate (OCR) was measured in SKNBE(2) (**A**–**C**) and KELLY cells (**D**–**F**). Cells were treated for 24 h with MET (1 mM to 10 mM) or vehicle (CTRL). The scatter plot of mitochondrial respiration indicates basal (**B**,**E**) and maximal respiration (**C**,**F**). Values represent mean ± SD of two independent experiments normalized to the DNA content per cell (CyQUANT). One-way ANOVA followed by Dunnett’s multiple comparison test; *p*-value **** *p* < 0.0001. Graphs (**A**,**D**) are representative for one of two independent experiments.

To determine if MET, as an inhibitor of mitochondrial complex I, could affect the respiration of MYCN-amplified NB cells, the SKNBE(2) and KELLY cells were treated with increasing concentrations of MET, and the oxygen consumption rate (OCR) was measured (Figure 1A,D). MET reduced both the basal (Figure 1B,E) and maximal respiration (Figure 1C,F) in a concentration-dependent manner. In the KELLY cells, 1 mM MET reduced the basal and maximal respiration to 31.4% and 38%, respectively. In the SKNBE(2) cells, however, the same concentration of MET reduced the basal and maximal respiration to 58.2% and to 51.4%, respectively, suggesting a higher sensitivity of the KELLY cells to MET.

**Figure 2:**
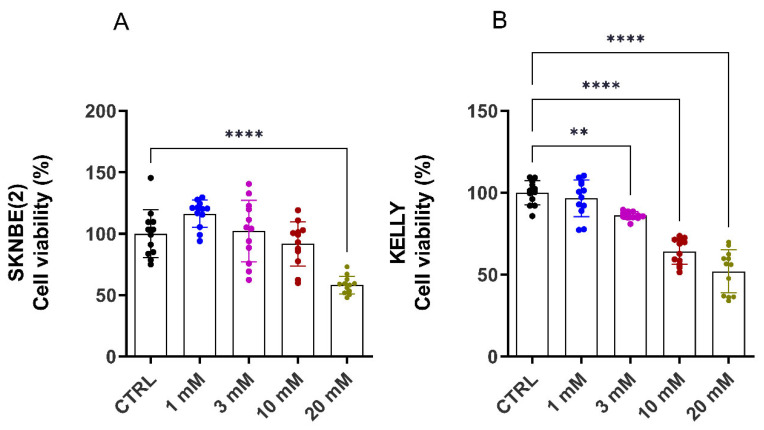
Effect of MET treatment (1–20 mM) for 72 h on cell proliferation of MYCN-amplified NB cell lines. Cell proliferation of SKNBE(2) (**A**) and KELLY (**B**) cells is indicated as percentage of CTRL as mean ± SD; statistical significance was determined by a one-way ANOVA with Dunnett’s multiple comparison test; *p*-value ** *p* < 0.01, **** *p* < 0.001 (*n* = 12 from 3 independent experiments).

To determine if the reduction in respiration had a direct effect on proliferation, the NB cells were treated with MET (1–20 mM) for 72 h. The anti-proliferative effect of MET was more pronounced in the KELLY (3 mM) versus the SKNBE(2) cells (20 mM) (Figure 2). This suggests that the KELLY cell line has a greater reliance upon mitochondrial respiration to drive cellular proliferation.

### 3.2. MET Enhanced the Anti-Tumor Effect of a KD and Low-Dose Chemotherapy on MYCN-Amplified NB Xenografts

**Figure 3:**
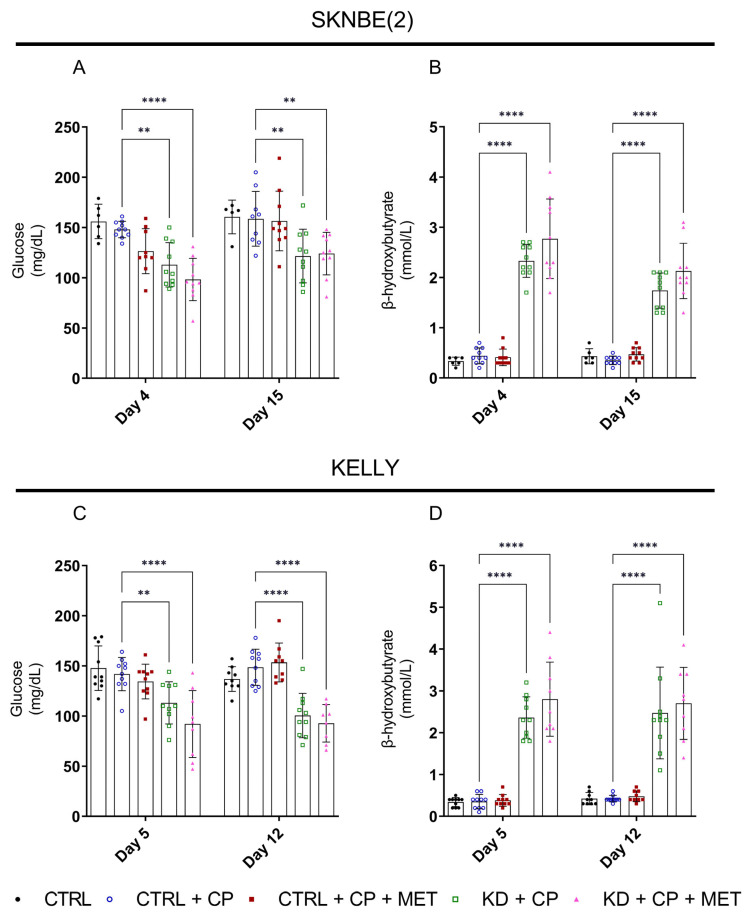
Effects of a KD (ketogenic ratio 8:1), MET (100 mg/kg administered by oral gavage), and low-dose CP (13 mg/kg/day in SKNBE(2) and 20 mg/kg/day in KELLY administered via drinking water) on blood glucose (**A**,**C**) and β-hydroybutyrate levels (**B**,**D**) in SKNBE(2) and KELLY xenograft-bearing mice. Statistical significance was evaluated using two-way ANOVA mixed effect analysis (Dunnett). Data are represented as mean ± SD. *p*-value ** *p* < 0.01, **** *p* < 0.0001 compared to CTRL + CP (*n* = 6–10).

We previously reported that a KD in combination with low-dose, cytotoxic CP slowed tumor growth in MYCN-amplified SKNBE(2) xenografts [19]. To enhance the anti-tumor efficacy of KD + CP, with a view to exploit the OXPHOS capacity of MYCN-amplified cells [8], we also treated NB-xenograft-bearing mice with a triple therapy combination of KD, CP, and MET. The triple therapy lowered plasma glucose in the SKNBE(2) and KELLY xenograft-bearing mice (Figure 3A,C). The levels of β-hydroxybutyrate increased in all of the KD groups, confirming that the KD induced ketosis in our xenograft models (Figure 3B,D). Moreover, the different therapy regimens did not induce any adverse effects in tumor-bearing mice, and their body weight was constant throughout therapy (Figure S4), confirming that the therapy regimens were well-tolerated.

**Figure 4:**
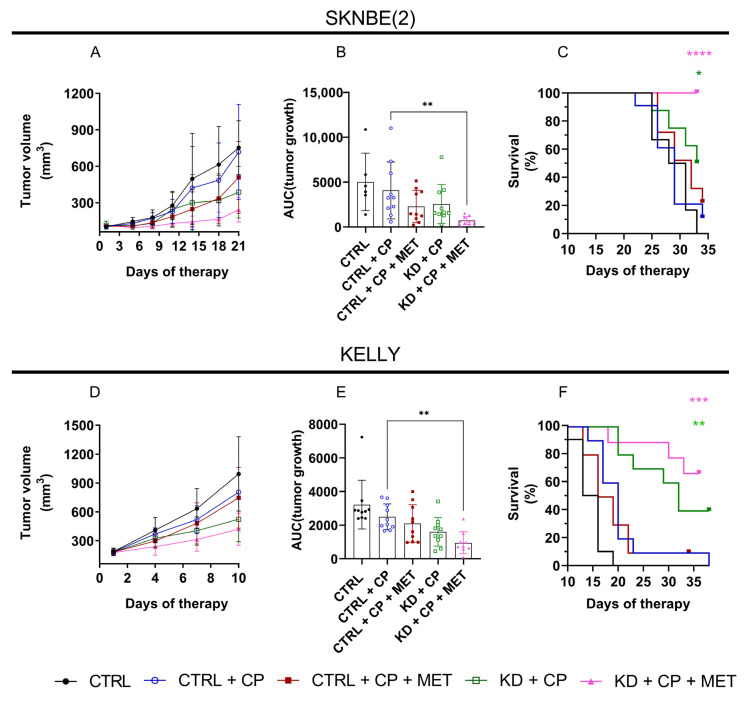
Effects of a KD (ketogenic ratio 8:1), MET (100 mg/kg administered by oral gavage), and low-dose CP (13 mg/kg/day in SKNBE(2) and 20 mg/kg/day in KELLY-bearing mice via drinking water) on the growth of SKNBE(2) and KELLY xenografts. Mean tumor volume ± SD is shown for each group until the first CTRL mouse tumor reached termination size (**A**,**D**); for statistical analysis of tumor growth data shown in (**A**,**D**), the area under the growth curve (AUC) was calculated for every mouse (**B**,**E**). AUCs are shown as mean ± SD. Statistical significance was determined by one-way ANOVA with Dunnett’s multiple comparisons. Survival analysis in SKNBE(2) (**C**) and KELLY (**F**) bearing mice was determined with log rank (Mantel–Cox) compared to CTRL + CP; *p*-value * *p* < 0.05; ** *p* < 0.01; *** *p* < 0.001; **** *p* < 0.0001 (*n* = 6–10).

Although MET in combination with CP did not reduce tumor growth in both xenograft models, the triple therapy consisting of KD, CP, and MET significantly reduced tumor growth (Figure 4B,E) and enhanced survival (Figure 4C,F) in both the SKNBE(2) and KELLY tumor-bearing mice. Individual growth curves of the xenografts treated with the different therapy regimens are presented in Figure S3.

### 3.3. Metabolic Peptide Levels in Plasma of MYCN-Amplified NB-Bearing Mice

Inflammation is a crucial factor driving tumor progression, by orchestrating fundamental neoplastic processes such as cell proliferation, survival, and migration [38]. Since both the KD and MET have been shown to regulate inflammation [39,40], an altered systemic inflammatory state may explain, in part, the anti-tumor effect observed upon triple treatment of the NB xenografts. Thus, ten different inflammatory markers (IL-1β, IL-2, IL-4, IL-5, IL-6, IL-12p70, IL-10, IFN-γ, KC/GRO, TNF-α) were measured in plasma samples of SKNBE(2)-bearing mice. Overall, these inflammatory factors were not affected by the different therapy regimens (Figure S5).

**Figure 5:**
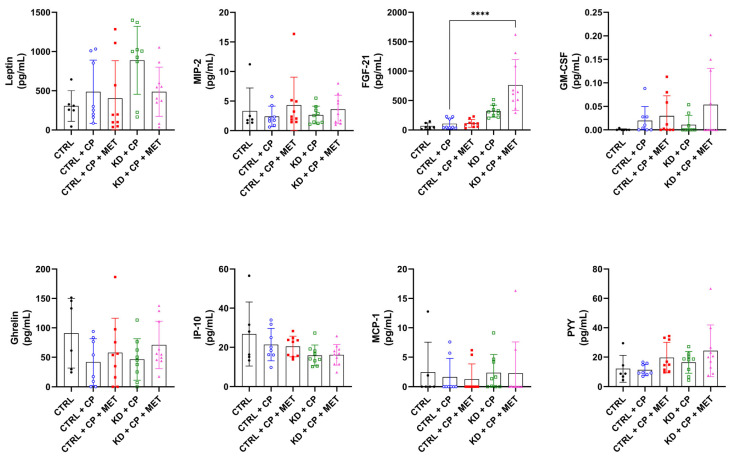
Effect of a KD, MET, and CP on the concentration of metabolic peptides (leptin, MIP-2, FGF-21, GM-CSF, ghrelin, IP-10, MCP-1, and PYY) in plasma of SKNBE(2) xenograft-bearing mice. Statistical significance between CTRL + CP and the other therapy groups was determined by one-way ANOVA with Dunnett´s multiple comparison test. Data are represented as mean ± SD; *p*-value **** *p* < 0.0001; (*n* = 8–10).

Since NB progression may also be affected by metabolic perturbations induced by the triple treatment, we measured the plasma levels of several metabolic peptides conceivably involved in NB progression, including leptin, MIP-2, FGF-21, GM-CSF, ghrelin, IP-10, MCP-1, and PYY (Figure 5). FGF-21 was the only metabolic peptide augmented by the triple therapy.

### 3.4. RNA-Seq Analysis and GO Enrichment Analysis in SKNBE(2) Tumor Tissue

**Figure 6:**
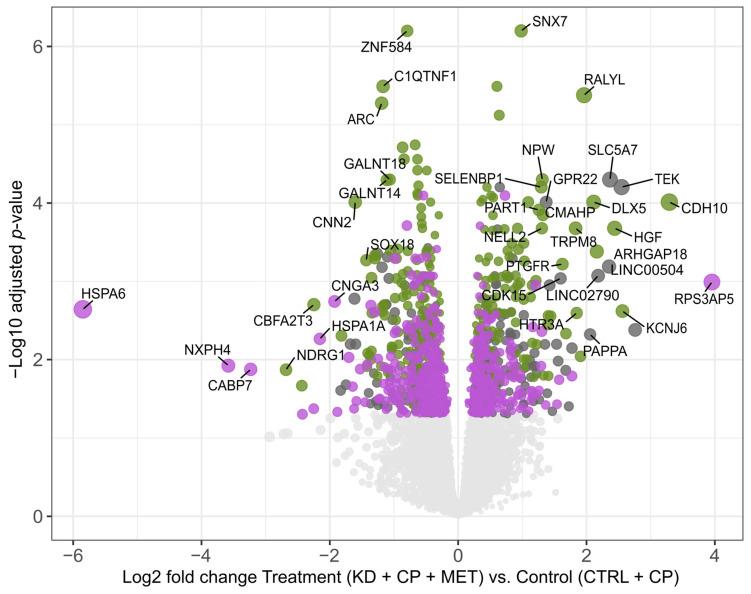
Volcano plot of differential gene expressions comparing triple treatment (KD + MET + CP) and control (CTRL + CP) conditions. Genes with significantly differential expression (adjusted *p*-value < 0.05) are classified into three categories: (1) KD-driven (dark grey dots); (2) additive effect (green dots)—significantly regulated in both combined treatment KD + MET + CP vs. CTRL + CP and KD + CP vs. CTRL + CP comparisons, with larger absolute fold changes in the presence of MET; (3) exclusive (purple dots)—differentially regulated only in the combined treatment KD + MET + CP vs. control comparison but not in KD + CP vs. CTRL + CP.

To gain a better understanding of which metabolic pathways are altered by the combination of the triple KD + CP + MET treatment, we performed bulk RNA-seq analysis on tumor samples from the SKNBE(2) xenografts. Principal component analysis (PCA) revealed a clear separation between samples obtained from CTRL- or KD-fed animals (Figure S6), suggesting that diet is the main driver of the inter-sample variability in gene expression. The RNA-seq analysis identified 326 differentially expressed genes due to the combined effect of KD and MET (i.e., additive effect), and 651 genes exclusively regulated by their combination (i.e., exclusive effect). HSPA6 was the most downregulated gene exclusively by the combination of KD, CP, and MET compared to the CTRL + CP group (Figure 6).

**Figure 7:**
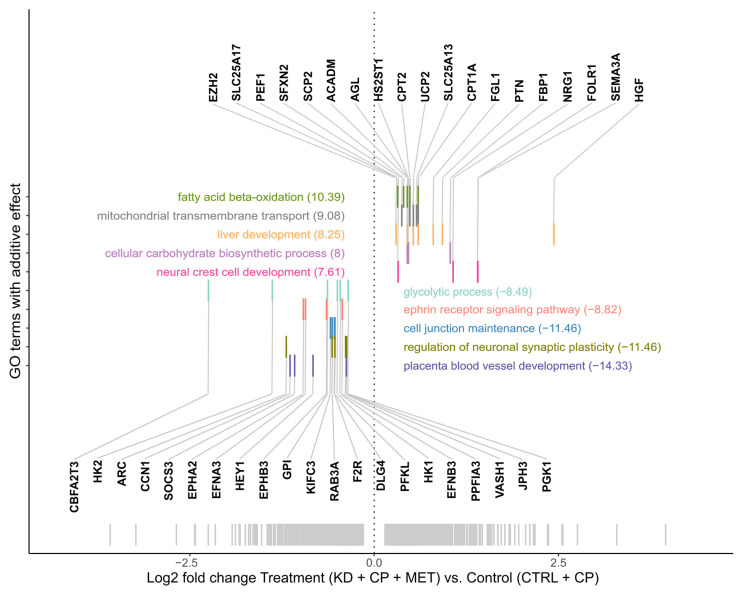
Gene ontology (GO) enrichment analysis for genes with additive effects (green dots in volcano plot) (*n* = 326, adjusted *p*-value < 0.05). Enriched genes and the corresponding ten most enriched GO biological processes are highlighted in the same color. Fold changes of GO enrichment analysis are shown next to the respective pathways. Genes found in several pathways are shown in one bar with different corresponding color codes. The remaining significantly differentially expressed genes are highlighted as grey barcodes on the bottom (Log2 FC triple treatment KD + MET + CP vs. CTRL + CP).

Notably, GO enrichment analysis revealed that fatty acid ß-oxidation, including solute carrier family 25 member 17 (SLC25A17), CPT1A, sterol carrier protein 2 (SCP2), acyl-CoA dehydrogenase medium chain (ACADM), and carnitine palmitoyltransferase 2 (CPT2) transcripts, was the most upregulated process by both KD groups (KD + CP + MET and KD + CP) compared to the CTRL + CP group, and MET augmented this effect (Figure 7, Tables S2 and S3).

Moreover, the genes DnaJ heat shock protein family (Hsp40) member B1 (DNAJB1), heat shock transcription factor 1 (HSF1), heat shock protein family A (Hsp70) member 1B (HSPA1B), and heat shock protein family A (Hsp70) member 1A (HSPA1A) involved in the “negative regulation of inclusion body assembly” were the genes most downregulated exclusively by the triple combination of KD, CP, and MET, but not by the combination of KD and CP, compared to CTRL + CP group (Table S5).

### 3.5. Triple Therapy with KD, CP, and MET Augmented the Expression of CPT1A

**Figure 8:**
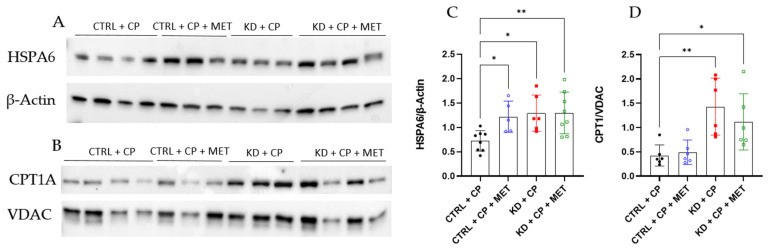
Western blot analysis of HSPA6 (**A**,**C**) and CPT1A (**B**,**D**) of SKNBE(2) xenografts. The relative expressionx of HSPA6 and CPT1A were normalized to β-actin and VDAC, respectively. Statistical significance was evaluated using one-way ANOVA followed by Dunnett’s multiple comparison test; *p*-value * *p* < 0.05, ** *p* < 0.01 (*n* = 5–8).

HSPA6 was the most downregulated gene by the triple therapy. Although the role of HSPA6 in cancer progression needs to be clarified [41], its expression has been shown to correlate with malignant progression in gliomas [42]. Therefore, we evaluated if the triple treatment also induced the downregulation of HSPA6 at the protein level. Surprisingly, Western blot analysis revealed that HSPA6 expression was in fact increased in all of the therapy groups (Figure 8A,C). As it was shown in the GO enrichment analysis, fatty acid β-oxidation was the most upregulated pathway by both KD groups (KD + CP + MET and KD + CP) compared to the CTRL + CP group, and MET increased this effect (Figure 7 and Table S2). Thus, we evaluated if the protein expression of CPT1A, a crucial enzyme involved in fatty acid β-oxidation, paralleled its upregulation at the protein level. Indeed, CPT1A protein expression was augmented by both therapies, KD + CP and KD + CP + MET, although the latter did not further enhance its expression, which was evident at the transcript level. The protein levels of CPT1A were normalized to the VDAC, a marker of mitochondrial content (Figure 8B,C). The unaltered VDAC expression confirmed that mitochondrial content was not affected by the different therapy regimens.

CPT1A is a rate-limiting enzyme for the β-oxidation of long-chain fatty acids in mitochondria. Therefore, a higher expression of CPT1A could indicate a greater uptake of long-chain fatty acids into mitochondria. The post-translational regulation of CPT1A induced by malonyl-CoA, the product of a reaction requiring the enzyme acetyl-CoA carboxylase (ACC), is a well-acknowledged mechanism that modulates this activity [43]. Therefore, we determined whether the higher CPT1A activity induced by the inhibition of ACC might affect mitochondrial energy metabolism and cellular respiration in NB cells. The SKNBE(2) and KELLY cells were treated with the ACC inhibitor PF 05175157 for 72 h, and the oxygen consumption rate (OCR) was measured using the Seahorse XF Cell Mito Stress Test.

PF 05175157 reduced basal respiration in both cell lines, whereas maximal respiration was only lower in the KELLY cells (Figure S7). The 33 µM PF 05175157 decreased cell proliferation of the SKNBE(2) cells, with no effect observed in the KELLY cells (Figure S8). Taken collectively, while the inhibition of ACC alone is not sufficient to disrupt NB cell growth, we cannot preclude the impact of upregulating CPT1A in vivo.

### 3.6. Effect of KD, CP, and MET on the Microbiota of MYCN-Amplified NB Xenografts

**Figure 9:**
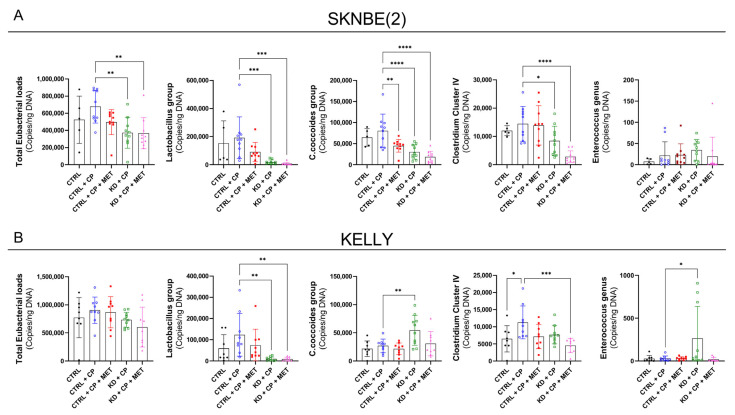
Effect of KD, CP and MET on gut microbiota of SKNBE(2) (**A**) and KELLY (**B**) xenograft-bearing mice. Statistical significance between (copies/ng DNA) of CTRL + CP and the other treatment groups was evaluated using one-way ANOVA with Dunnett´s multiple comparison test. Data are represented as mean ± SD; *p*-value * *p* < 0.05, ** *p* < 0.01, *** *p* < 0.001, **** *p* < 0.0001 (*n* = 5–10).

The role of the gut microbiota in cancer development is well-established [44,45]. MET has been associated with the maintenance of a healthy gut microbiome [46], whereas the role of KD on the microbiota is not fully elucidated [47]. To reveal if different therapy regimens could alter the gut microbiota composition, the total eubacterial loads and the abundance of the main commensal gut bacterial phyla such as Enterobacteriaceae, *Enterococcus* genus, *Lactobacillus* group, *Bifidobacterium* genus, *Bacteroides*/*Prevotella* spp., *Clostridium coccoides* group, *Clostridium* cluster IV, and *Mouse Intestinal Bacteroides* were quantitatively surveyed using qRT-PCR. Bacteria from the *Lactobacillus* group were reduced in all of the KD groups in both xenografts. Conversely, the total eubacterial loads, as well as the gene numbers of the *Clostridium coccoides* group and *Clostridium* cluster IV *Enterococcus* genus, were differentially affected by the therapy regimens in the SKNBE(2) and KELLY xenografts-bearing mice (Figure 9). The bacterial groups that were not affected by their respective regimens are reported in Figure S9. Hence, these differences between the two NB models suggest that not only is the gut microbiota altered by the therapy regimen, but also by the intrinsic properties of the tumor.

## 4. Discussion

MYCN-induced metabolic reprogramming is a hallmark of NB [9], and has been shown to promote NB growth and proliferation by rewiring glutamine [48,49], glucose [50,51,52], and oxidative metabolism [8]. However, all efforts to date that aimed at either directly or indirectly targeting MYCN have not translated into clinical use [5].

In the present study, we demonstrated that MET inhibited oxidative metabolism of MYCN-amplified NB cells by blunting cellular respiration. Despite the striking effects on respiration, this was not paralleled by similar reductions in cell proliferation, pointing to the metabolic flexibility of MYCN-amplified NB cells to sustain growth when challenged by metabolic impairments. This phenomenon is in agreement with other studies showing that the metabolic environment may determine sensitivity of cancer cells to MET [53,54,55,56]. In support of this notion, we observed either a partial disruption to tumor growth, or no response at all, in MYCN-amplified NB xenografts treated with MET alone (Figure S2) or in combination with CP (Figure 4). In contrast to our findings, Kumar at al. showed that MET alone was capable of inhibiting the growth of MYCN-amplified and non-amplified NB tumors, including SKNBE(2) xenografts [26]. However, when we combined MET with a KD and low-dose CP, there was an appreciable disruption of tumor growth associated with improved survival in mice bearing SKNBE(2) and KELLY xenografts. Although the in vitro data demonstrated a higher sensitivity of KELLY cells to MET, metabolic therapy in vivo induced a stronger survival advantage in the SKNBE(2) compared to KELLY xenografts-bearing mice. Interestingly, the growth of KELLY tumors appeared to be faster compared to the SKNBE(2) tumors, suggesting higher anabolic demands to sustain tumor growth. Despite the aggressiveness of this model, both KD + CP and KD + CP + MET were able to counteract the rapid tumor growth of the KELLY xenografts, extending mouse survival significantly.

It is noteworthy that in the present study as well as in previous studies [26,57], the concentration of MET required to reduce cell respiration in vitro (millimolar) is a magnitude of order higher than the plasma levels of MET detected in vivo (micromolar) [58]. Although MET can accumulate in tumor and normal lung tissue, the concentrations are still 100- to 1000-fold lower than effective doses in the in vitro studies [59]. Moreover, MET accumulation has been shown to be associated with organic cation transporter 2 (OCT2) protein expression, and may predict anti-cancer effects in vivo [60]. As previously mentioned, the heterogeneous tumor microenvironment appears to dictate the sensitivity to MET. In that regard, the nutrient composition (e.g., glucose concentration of 7.8 mM) and growth factor abundance of standard cell culture media evidently fails to faithfully recapitulate the nutritional conditions of poorly vascularized tumors [61]. Indeed, in tumor interstitial fluid, the glucose levels of mice treated with a KD were reported to range between 1.3 and 4.5 mM [17]. Chandel et al. suggested that the use of millimolar concentrations of MET in vitro cannot be used to predict its biological activity in vivo or in the clinic [58]. This has been confirmed by Wheaton and colleagues, who showed that plasma concentrations of MET eliciting an anti-tumor effect through inhibition of OXPHOS are in the micromolar range in a murine model [22]. Altogether, these data support the fact that the reduction in tumor growth observed in our xenograft models is attributable to the addition of MET to the KD and CP, and conceivably a result of MET-induced inhibition of complex I.

Inflammation is known to drive crucial processes related to tumor progression [62]. Despite this, our triple therapy approach did not alter the levels of systemic pro-inflammatory factors, suggesting that changes in the inflammatory status are unlikely to explain the anti-tumor effect observed in our NB xenograft models. It should be pointed out, however, that CD-1 immuno-deficient mice do not possess T-lymphocytes. Hence, the inflammatory response measured in the present murine xenograft models may not accurately reflect that of an immunocompetent individual. Screening of metabolic peptides revealed that the triple therapy increased the level of FGF-21. The hepatokine FGF-21 has been shown to be therapeutically beneficial for the treatment of metabolic diseases via reducing fat mass, hyperglycemia, insulin resistance, dyslipidemia, and non-alcoholic steatohepatitis in rodents and non-human primates [63]. Furthermore, FGF-21 regulates fatty acid metabolism by promoting lipolysis, fatty acid oxidation, and thermogenic energy dissipation [64,65,66], and is crucial for the full adaptation to a KD [67]. It is noteworthy that one study demonstrated that the anti-tumor effect of a KD was FGF-21-independent in a model of Lewis lung carcinoma [68].

Thus, to reveal how different therapy regimens and metabolic alterations may alter tumor metabolism and pinpoint potential mechanisms underpinning the anti-cancer effect(s) of the triple combination of KD, CP, and MET, we performed RNA-seq analysis on the more therapeutically-responsive SKNBE(2) xenografts. The RNA-seq analysis demonstrated that *HSPA6* was the most downregulated gene by the triple therapy, although this did not correspond to a reduction at the protein level, which actually increased. Although the role of HSPA6 in cancer progression needs to be clarified [41], its expression was previously correlated with malignant progression in glioma [42]. GO enrichment analysis indicated that fatty acid β-oxidation was the most upregulated process by both KD groups (KD + CP + MET and KD + CP) compared to the CTRL + CP group, and MET augmented this effect. This finding was supported by a higher expression of CPT1A at the protein level.

The role of β-oxidation in cancer development remains controversial, yet several studies have demonstrated a pro-tumor effect via inhibition of the apoptotic cascade, sustaining OXPHOS, and preserving mitochondrial function [69,70,71,72,73,74]. Indeed, Tao et al. recently showed that higher fatty acid uptake can sustain the growth of MYCN-amplified NB [75]. In our hands, we observed a survival advantage in xenografts treated with a KD, although the most prominent effect was induced by the triple combination of KD, CP, and MET. GO enrichment analysis revealed a higher expression of genes related to β-oxidation in tumors treated with KD. This may indicate that the KD-induced glucose limitation promotes a greater dependency on β-oxidation. In that regard, there may be an elevated demand to recycle cofactors derived from β-oxidation, i.e., NADH and FADH2, which are unlikely to be efficiently re-oxidized by complex I due to exposure to MET. Thus, NB cells treated with the triple therapy may not be capable of maintaining a balanced redox state to thereby sustain the high-energy demands of cancer cells. Liu et al. demonstrated that fatty acid oxidation (FAO) could fuel the tricarboxylic acid cycle (TCA cycle) in the condition of glucose deprivation. Interestingly, cancer cells were sensitive to MET when FAO was augmented, likely due to a slower capacity of FAO to fuel the TCA cycle compared to glucose or glutamine [76]. The metabolic intermediates of the TCA cycle are required for the production of building blocks for nucleotide, lipid, and amino acid synthesis to sustain the greater anabolic and energetic demands of cancer cells [6,77]. Thus, a hindered TCA cycle may cause a survival disadvantage in cancer cells. Moreover, Yang et al. demonstrated that the combination of a KD and cytotoxic chemotherapy significantly increased NADH, suppressed tumor glucose levels, and TCA intermediates, ultimately leading to a substantially improved survival in a pancreatic cancer model [18]. Together, these data support the hypothesis that the triple therapy might hamper the capacity of MYCN-amplified NB to efficiently utilize β-oxidation due to the inhibition of complex I, compromising the ability of mitochondria to sustain the elevated energy and anabolic demands of MYCN-amplified NB.

This unique metabolic state induced by the combination of KD, CP, and MET might explain the augmented anti-tumorigenic effect observed in MYCN-amplified NB xenografts. Although MET alone may elicit anti-cancer activity in some contexts, it appears to have the greatest anti-tumor potential as an adjuvant therapy. The present study examined MYCN-amplified NB xenografts established in CD-1 nude mice that are unable to produce T-cells, creating immune responses that may not accurately reflect an immunocompetent individual. However, KDs have been demonstrated to inhibit tumor growth by enhancing the immune response in different immunocompetent models [78,79], suggesting that an improved immune response may augment the anti-tumor effect of the metabolic therapies applied in our model. In that regard, KDs have already shown promising results as adjuvant therapy to immunotherapies [80,81]. Our study adds to the increasing evidence that the multi-modal metabolic targeting of cancer is a promising adjuvant therapy to tackle MYCN-amplified NB.

## Figures and Tables

**Table 1:** Murine pro-inflammatory factors and metabolic peptides measured via MSD.

V-Plex (A)	U-Plex (B)
Interferon-γ (IFN-γ)	Leptin
Interleukin-1β (IL-1β)	Macrophage inflammatory protein-2 (MIP-2)
Interleukin-2 (IL-2)	Fibroblast growth factor-21 (FGF-21)
Interleukin-4 (IL-4)	Granulocyte-macrophage colony-stimulating factor (GM-CSF)
Interleukin-5 (IL-5)	Ghrelin
Interleukin-6 (IL-6)	Human interferon-inducible protein-10 (IP-10)
Interleukin-10 (IL-10)	Monocyte chemoattractant protein-1 (MCP-1)
Interleukin-12p70 (IL12p70)	Pancreatic peptide YY (PYY)
Keratinocyte chemoattractant (KC)/human growth-regulated oncogene (GRO) chemokine (KC/GRO)	
Tumor necrosis factor-α (TNF-α)	

## Data Availability

Raw RNAseq data and processed files were submitted to GEO NCBI (GSE236677).

## Supplementary Materials

The following supporting information can be downloaded at: https://www.mdpi.com/article/10.3390/metabo13080910/s1, Figure S1: Effect of CP (10–40 mg/kg) on growth of KELLY xenografts; Figure S2: Effect of MET (50, 100, 250 mg/kg) on growth of SKNBE(2) and KELLY xenografts; Figure S3: Effect of a KD (ketogenic ratio 8:1), MET (100 mg/kg administered by oral gavage) and low-dose CP (13 mg/kg/day in SKNBE(2) and 20 mg/kg/day in KELLY-bearing mice via drinking water) on the growth of SKNBE(2) and KELLY xenografts; Figure S4: Effect of a KD (ketogenic ratio 8:1), MET (100 mg/kg administered by oral gavage) and low-dose CP (13 mg/kg/day in SKNBE(2) and 20 mg/kg/day in KELLY-bearing mice via drinking water) on the body weight of single CD-1 nude mice bearing SKNBE(2) (A–E) and KELLY (G–K) NB xenografts; Figure S5: Effect of KD, MET and CP on the concentration of inflammatory markers in plasma of SKNBE(2) xenograft bearing mice at the day of termination; Figure S6: Principle component analysis (PCA) based on the whole transcriptome data, showing sample clustering for the different experimental groups; Figure S7: Oxygen consumption rate (OCR) measured in SKNBE(2) (A–C) and KELLY (D–G) cells; Figure S8: Effect on cell proliferation of MYCN-amplified NB cell lines treated with the ACC inhibitor PF 05175157; Figure S9: Effect of KD, MET and CP on the gut microbiome of SKNBE(2) (A) and KELLY (B) xenograft bearing mice at the day of termination. Table S1: Composition of the CTRL and Ketogenic diet; Table S2: Genes with significant differential expression (adjusted *p*-value < 0.05) upregulated in both combined treatment (KD + MET + CP) vs. control and (KD + CP) vs. control comparisons, with larger absolute fold changes in the presence of MET (additive effect); Table S3: Genes with significant differential expression (adjusted *p*-value < 0.05) downregulated in both combined treatment (KD + MET + CP) vs. control and (KD + CP) vs. control comparisons, with larger absolute fold changes in the presence of MET (additive effect); Table S4: Genes with significant differential expression (adjusted *p*-value < 0.05) differentially upregulated only in the combined treatment (KD + MET + CP) vs. control comparison but not in (KD + CP) vs. control; Table S5: Genes with significant differential expression (adjusted *p*-value < 0.05) differentially downregulated only in the combined treatment (KD + MET + CP) vs. control comparison but not in (KD + CP) vs. control.
