# Supplementary material for: Triple Therapy with Metformin, Ketogenic Diet, and Metronomic Cyclophosphamide Reduced Tumor Growth in MYCN-Amplified Neuroblastoma Xenografts
Source: Metabolites. 2023 Aug 3;13(8):910. doi: 10.3390/metabo13080910 (PMC10456943; doi:10.3390/metabo13080910)
Supplement: Supplementary file 1 [file metabolites-13-00910-s001.zip › metabolites-2521116-supplementary.pdf]

Supplementary Figures

Triple Therapy with Metformin, Ketogenic Diet and Metronomic Cyclophosphamide Reduced Tumor Growth in MYCN-Amplified Neuroblastoma Xenografts

Luca Catalano, Sepideh Aminzadeh-Gohari, Daniela D. Weber, Rodolphe Poupardin, Victoria E. Stefan, William J. Smiles, Julia Tevini, René G. Feichtinger, Sophia Derdak, Martin Bilban, Stefan Bareswill, Markus M. Heimesaat, Barbara Kofler

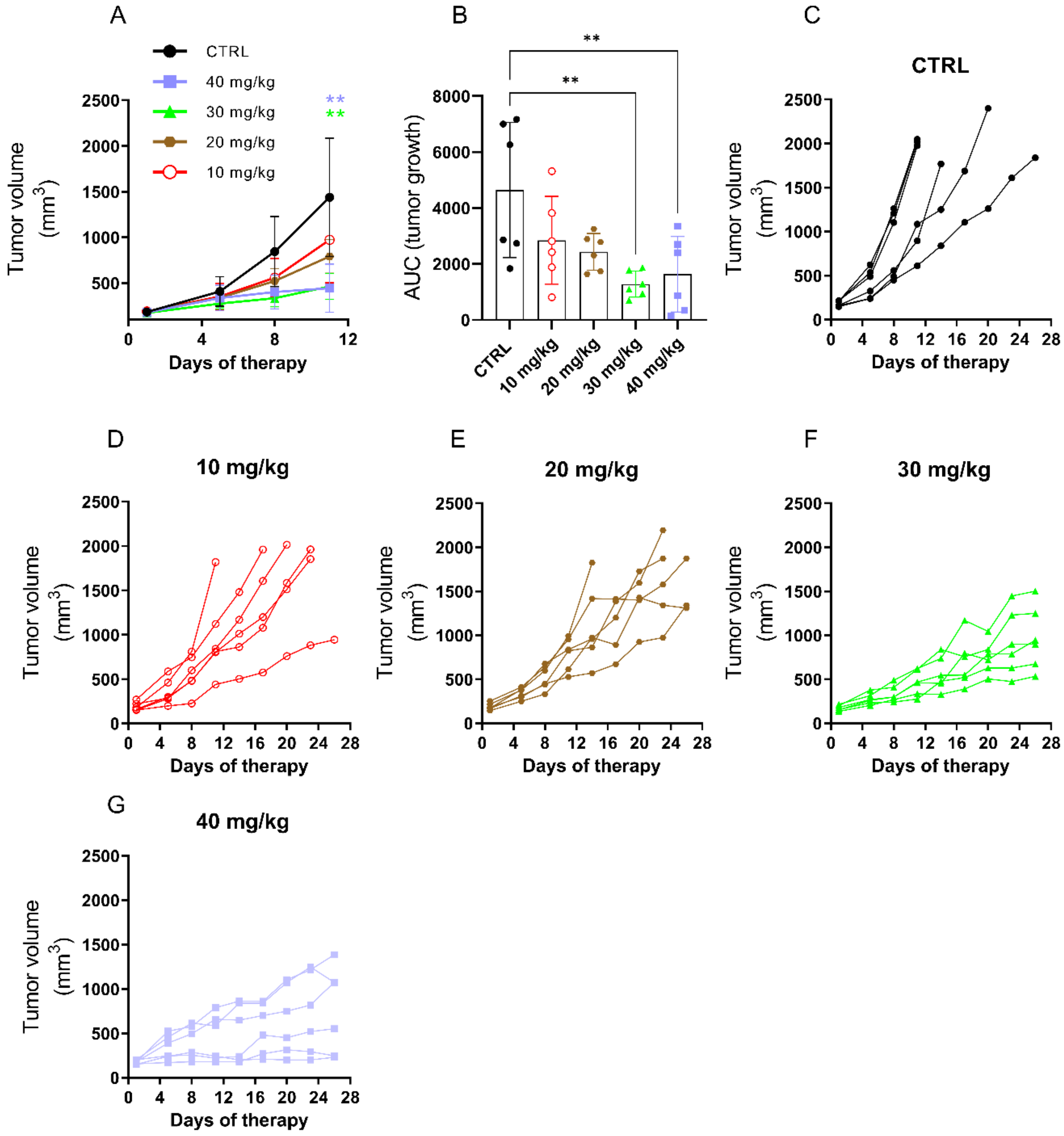

Figure S1. Effect of CP (10-40 mg/kg) on growth of KELLY xenografts. Mean tumor volume ± SD is shown for each group until the first CTRL mouse tumor reached the termination size (A); for statistical analysis of tumor growth data shown in A, the area under the growth curve (AUC) was calculated for every mouse. AUCs are shown as mean ± SD (B). Individual tumor growth of KELLY xenografts in single CD-1 nude mice treated with different concentrations of CP (C-G). Statistical significance was determined by a one-way ANOVA with Dunnett's multiple comparisons test; p-value **p<0.001 compared to CTRL (n=6).

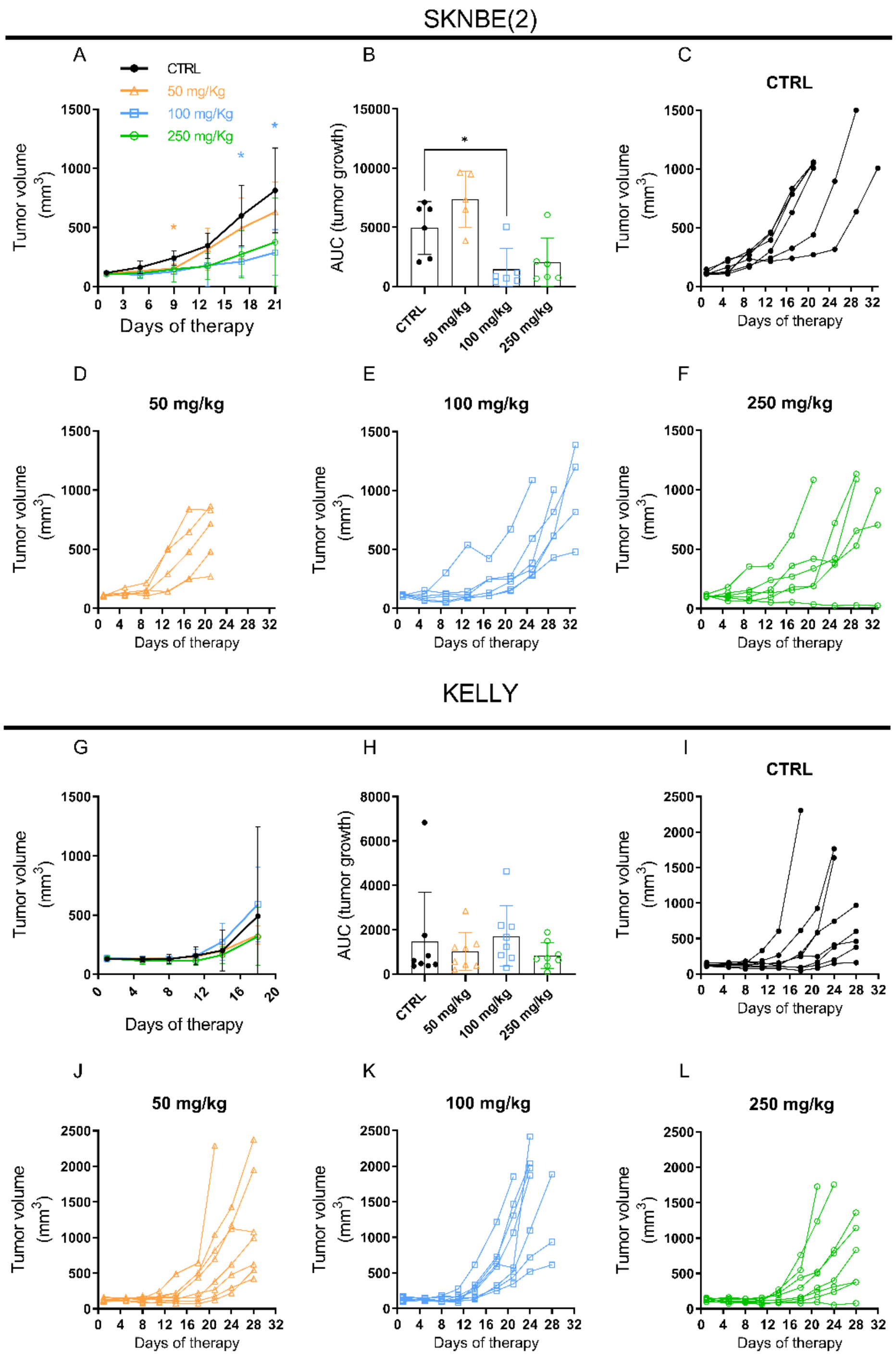

Figure S2. Effect of MET (50, 100, 250 mg/kg) on growth of SKNBE(2) and KELLY xenografts. Mean tumor volume ± SD is shown for each group until the first CTRL mouse tumor reached the termination size (A, G); for statistical analysis of tumor growth data shown in A and G, the area under the growth curve (AUC) was calculated for every mouse (B, H). AUCs are shown as mean ± SD. Tumor growth curves of individual SKNBE(2) (C-F) and KELLY (G-L) xenografts in CD-1 nude mice treated with different concentration of MET (50-250 mg/kg). Statistical significance was determined by a one-way ANOVA with Dunnett's multiple comparisons test; p-value *p<0.05 (n=6-8).

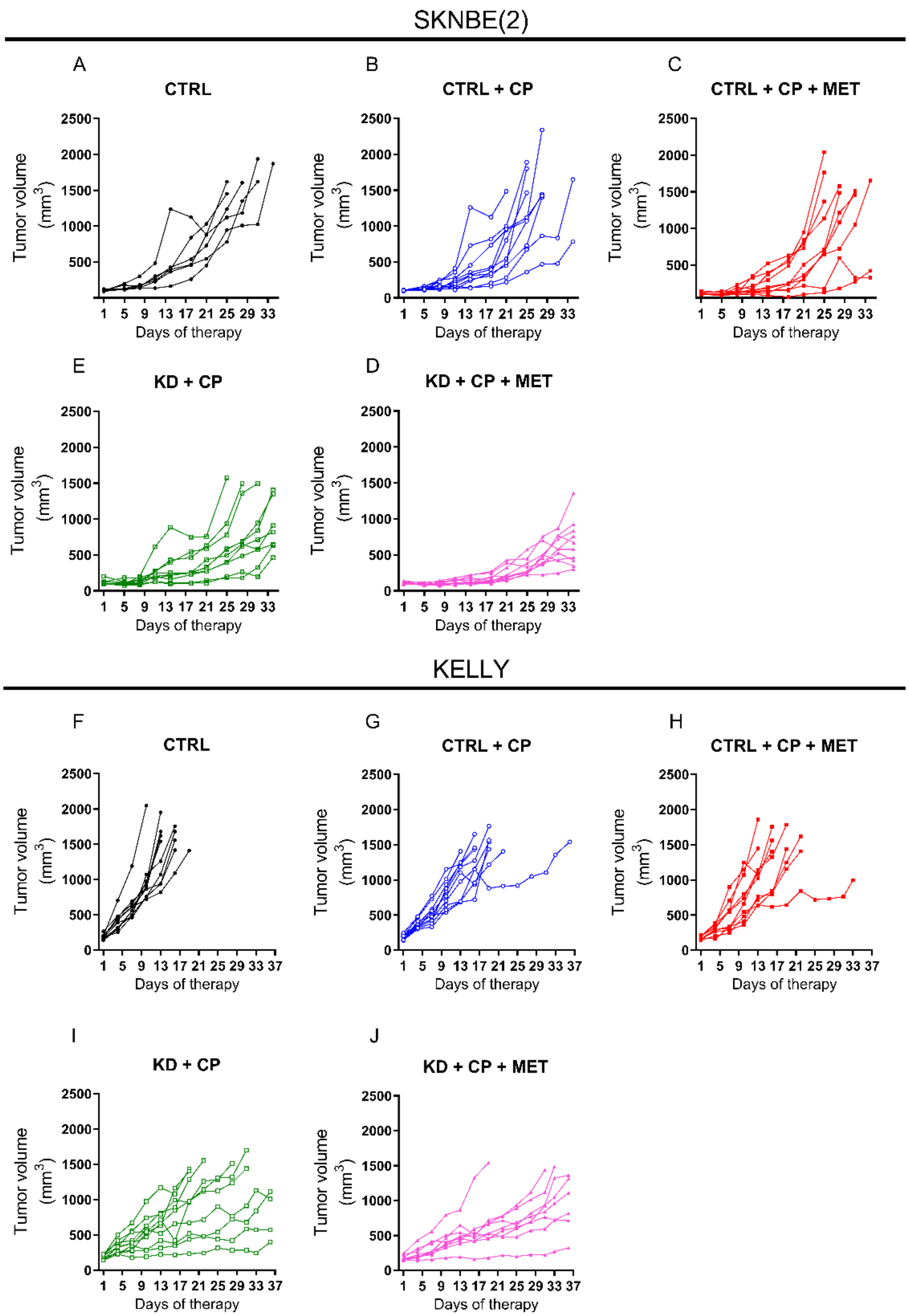

Figure S3. Effect of a KD (ketogenic ratio 8:1), MET (100 mg/kg administered by oral gavage) and low-dose CP (13 mg/kg/day in SKNBE(2) and 20 mg/kg/day in KELLY-bearing mice via drinking water) on the growth of SKNBE(2) and KELLY xenografts. Tumor growth curves of SKNBE(2) (A-E) and KELLY (F-J) xenografts in single CD-1 nude mice treated with different therapy regimens mentioned above (n=6-10).

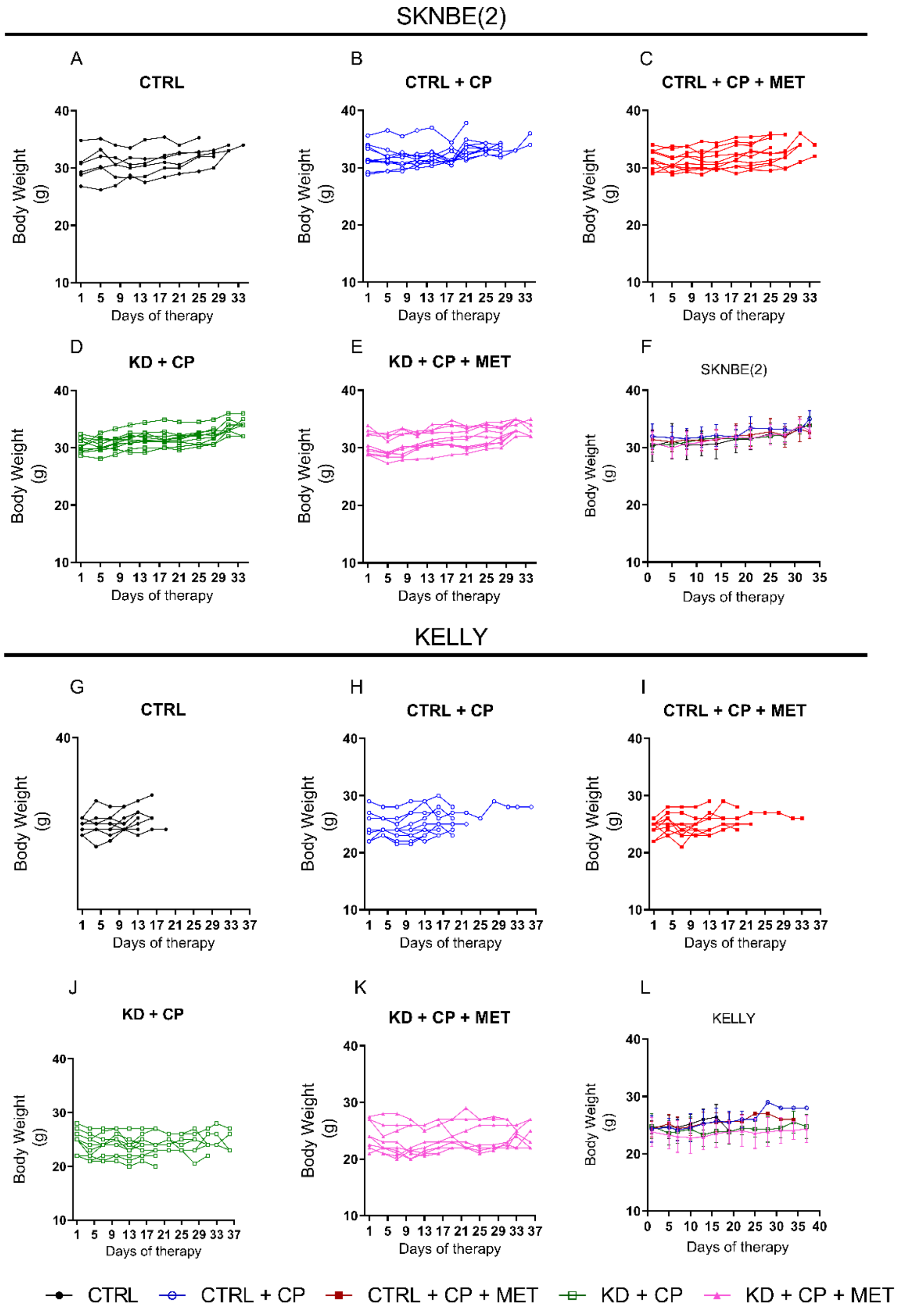

Figure S4. Effect of a KD (ketogenic ratio 8:1), MET (100 mg/kg administered by oral gavage) and low-dose CP (13 mg/kg/day in SKNBE(2) and 20 mg/kg/day in KELLY-bearing mice via drinking water) on the body weight of single CD-1 nude mice bearing SKNBE(2) (A-E) and KELLY (G-K) NB xenografts. Mean of body weight ± SD is shown for each group in SKNBE(2) (F) and KELLY (L) xenograft bearing mice (n=6-10).

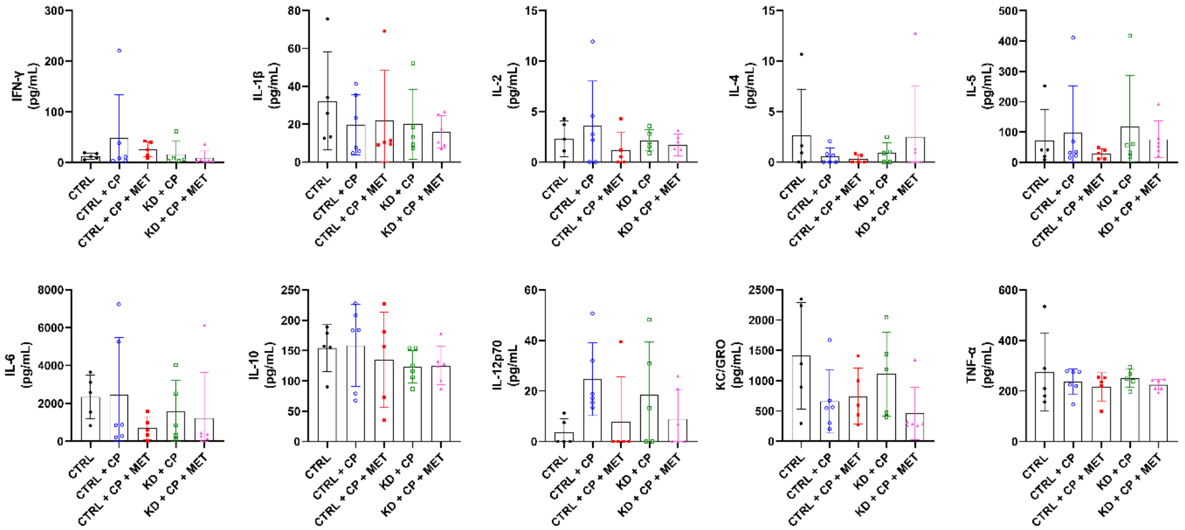

Figure S5. Effect of KD, MET and CP on the concentration of inflammatory markers in plasma of SKNBE(2) xenograft bearing mice at the day of termination. Statistical significance was evaluated using one-way ANOVA with Dunnets´s multiple comparison test. The single data points represent the mean of two replicates. Data are shown as mean ± SD (n=5-6).

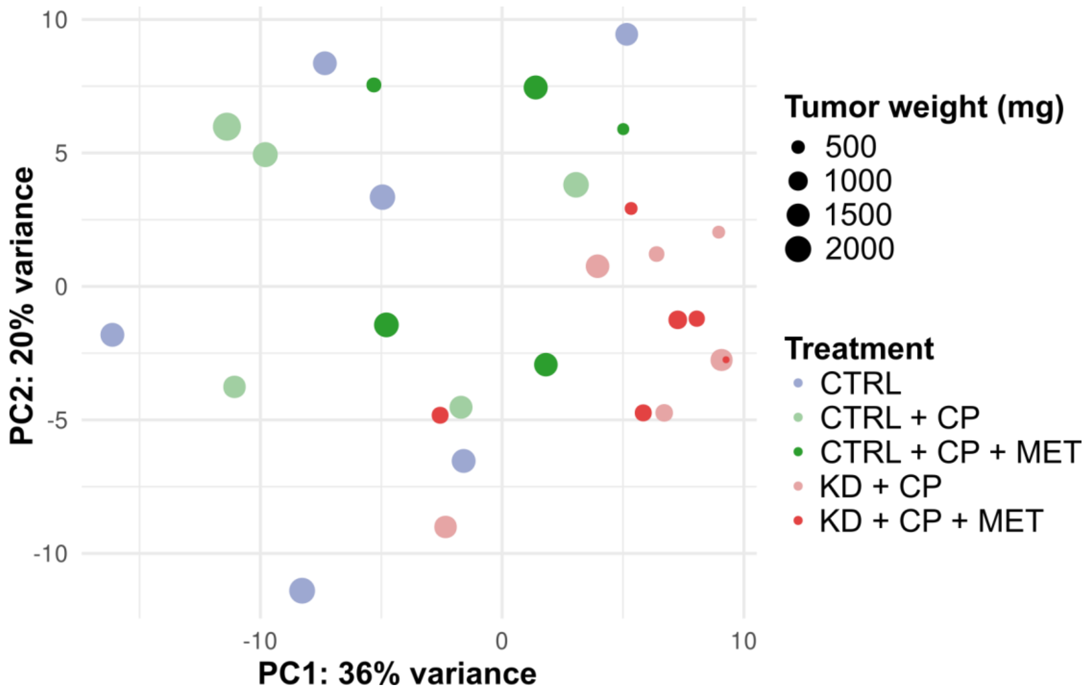

Figure S6. Principal component analysis (PCA) based on the whole transcriptome data, showing sample clustering for the different experimental groups. Size of the dots indicate the tumor end weight (mg). The KD treatment shows the biggest impact on the PCA clustering.

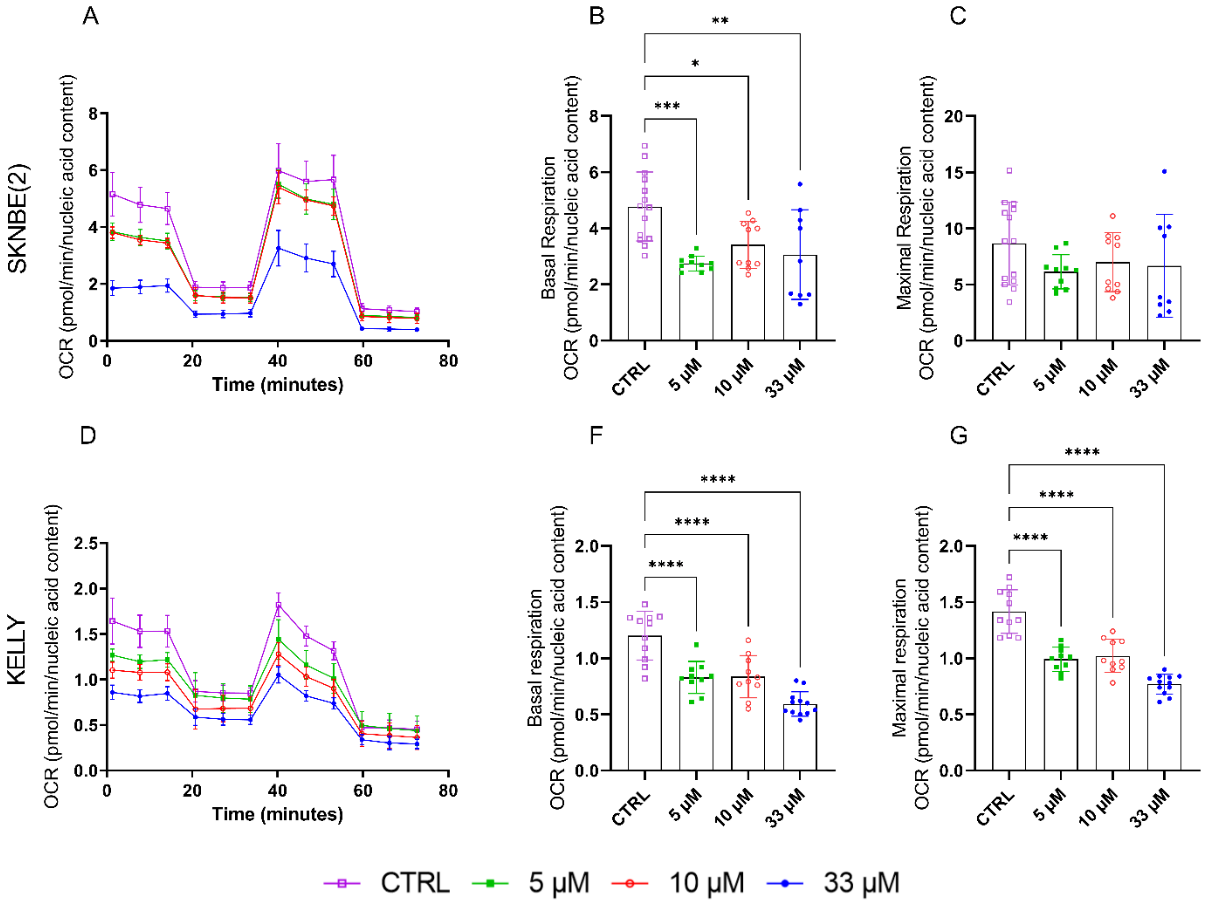

Figure S7. Oxygen consumption rate (OCR) measured in SKNBE(2) (A-C) and KELLY (D-G) cells. The cells were treated for 72h with PF05175157 (1 µM to 33 µM) or vehicle (CTRL). The scatter plot of the mitochondrial respiration are showing basal respiration (B, F) and maximal respiration (C, G). Values represent mean ± SD of two independent experments normalized to the DNA content per cell. One-way ANOVA followed by Dunnett's Multiple Comparison Test; *p<0.05, **p<0.01, ***p<0.001; ****p<0.0001. The graphs are representative for two independent experiments (A, D).

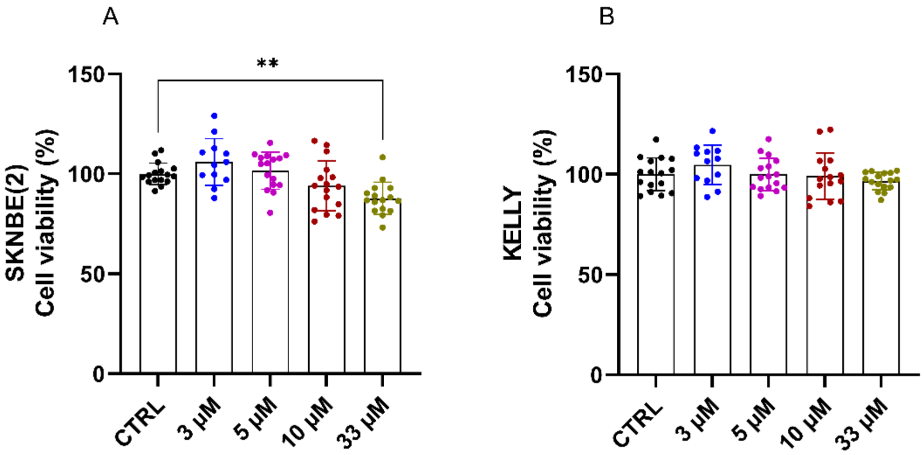

Figure S8. Effect on cell proliferation of MYCN-amplified NB cell lines treated with the ACC inhibitor PF 05175157. Cell proliferation of SKNBE(2) (A) and KELLY (B) cells is indicated as percentage of CTRL. Data are presented as mean ± SD of two independent experiments. One-way ANOVA followed by Dunnett's Multiple Comparison Test; **$p<0.001$, ($n=8$ from 2 independent experiments).

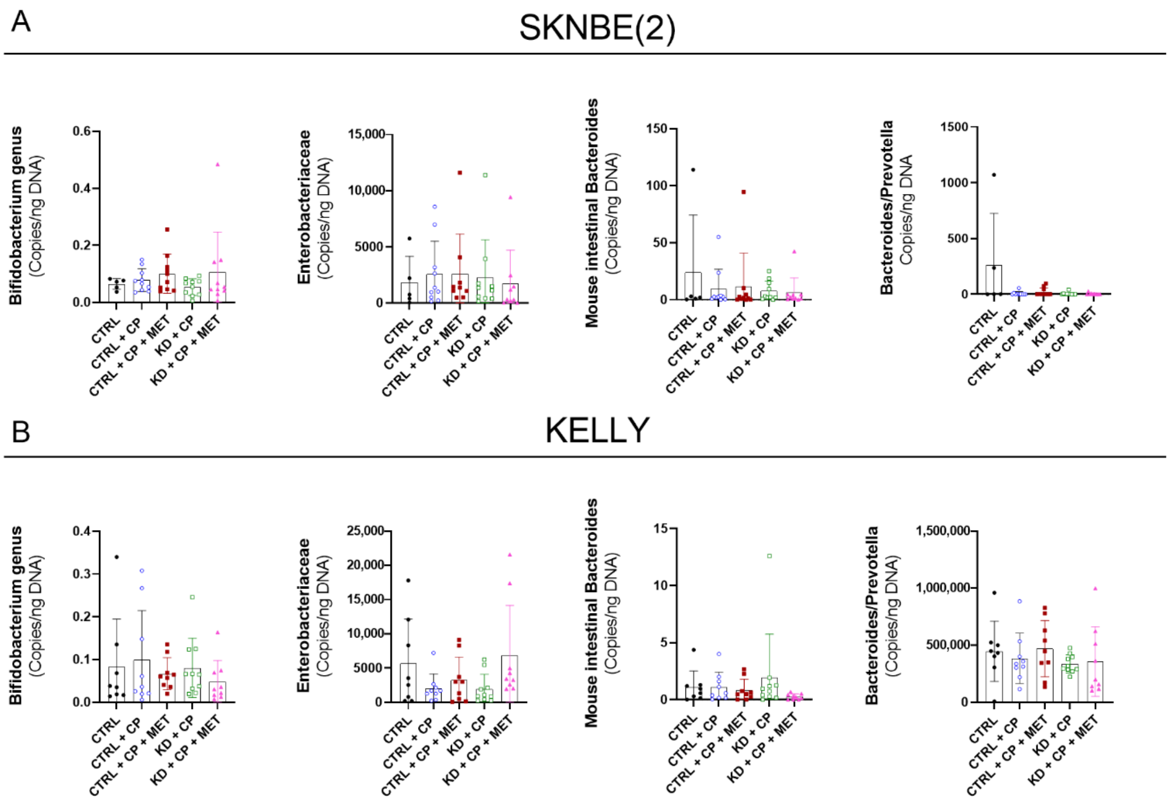

Figure S9. Effect of KD, MET and CP on the gut microbiome of SKNBE(2) (A) and KELLY (B) xenograft bearing mice at the day of termination. Statistical significance between (copies/ng DNA) of CTRL+CP and the other treatment groups was evaluated using one-way ANOVA with Dunnets´s multiple comparison test. Data are represented as mean ± SD, (n=5-10).
